# Differences in the clinical manifestations and short-term prognosis of acute cerebral infarction after exposure to Agent Orange

**DOI:** 10.1186/s40557-016-0137-9

**Published:** 2016-11-18

**Authors:** SangWoo Han, Inha Hwang, Seung Min Kim, Young Soon Yang, SangWon Ha, Jeong Ho Han, Tae Hwan Park

**Affiliations:** 1 Department of Neurology, Veterans Health Service Medical Center, Dunchon 2-dong, Gangdong-gu, Seoul 134-791 Korea; 2 Department of neurology, Seoul Medical Center, Seoul, Korea

## Abstract

**Background:**

Agent Orange (AO) is the code name for one of the herbicides and defoliants used in the Vietnam War. Studies conducted thus far show a significant correlation between AO and the occurrence of cardiovascular diseases. But there is little data on the association between AO and stroke, and limited studies have targeted patient groups exposed to AO.

**Method:**

Bohun medical center Institutional Review Board (IRB) approved the study. (ID: 341) We studied patients with acute ischemic stroke within 7 days of onset in VHS medical center and 4 other general hospitals. Among them, 91 consecutive patients with previous exposure to AO were evaluated. For controlled group, 288 patients with no history of AO exposure were chosen.

**Result:**

There were 49 (44.0 %) DM patient with a higher frequency in the exposure group (93 (32.3 %) in control *P* = 0.045). There were 6 (6.6 %) hyperlipidemia in exposure group and 69 (24.0 %) in control. (*P* < 0.002). Small vessel occlusion was the most common subtype (36, 39.6 %) in exposure group but in control group, the large artery atherosclesosis was (120, 41.7 %) (*P* = 0.014).

The NIHSS of the exposure group on admission showed lower scores (median values, 2 and 4, respectively; *P* = 0.003). The median mRS was 1 for the exposure group and 2 for the control group, at discharge and after 3 months. After 3 months of discharge, 55 (60.4 %) in the exposure group and 171 (59.4 %) in the control group showed below mRS 1 (*P* = 0.001).

**Conclusion:**

This study targeted patients who are Vietnam veteran. There is some difference in vascular risk factors and clinical manifestations suggest AO exposure has contributed to a certain extent to the stroke.

## Background

Agent orange (AO) is an organic chemical containing several toxic materialsused during the Vietnam War to improve visibility. Among these materials, 2,3,7,8-tetrachlorodibenzo- p-dioxin (TCDD), also known as dioxin and included in a small quantity, has the highest toxicity. TCDD is thought to be the cause of contemporary AO toxicity. The half-life of TCDD is 7.1–11.3 years, and therefore, its adverse effects result from accumulation in the human body [1].Human experiments on the effects of AO cannot be performed, and therefore,only animal experiments or epidemiological investigationsare possible. Apart from the differences between animals and humans, the limitation of animal experiments is that the average age span of animals is 2–3 years compared to the long half-life of TCDD. With regards to epidemiological investigations, there is little data of Vietnam veterans who have been exposed to AO, and there is no data regarding the amount of exposure to AO, which makes quantitative analysis impossible. Therefore, we can only obtain data from occupational, environmental, and case-controlled studies using similar substances and draw indirect inferences [2]. Studies conducted thus far show a significant correlation between AO and the occurrence of cardiovascular diseases, such as ischemic heart disease (IHD) and atherosclerosis and risk factors, such as diabetes mellitus (DM) and hypertension (HTN). Alternatively, stroke is assumed to be associatedwith AO as it shares many risk factors and pathogenic mechanismsfor cardiovascular diseases, but this has not been clearly demonstrated. There is little data on the association between AO and stroke, and limited studies have targeted patient groups exposed to AO [3].In this study, we aim to compare stroke patients with and without a history of AO exposure and study the differences in the clinical manifestations and short-term prognosis.

## Method

### Subjects

Our current study was a prospective investigation of acute ischemic stroke patients admitted to the VHS Medical Center between March 2008 and February 2010. We screened consecutive patients who (1) is Vietnam veterans officially confirmed by the government, (2) had acute ischemic stroke confirmed by initial MRI within 1 week after onset, and (3) had clinical follow-up for 3 months. Patients who had contraindications to MRI were excluded. Male control subjects who did not experience Vietnam war and aged 55–75 years were randomly selected from acute ischemic stroke patients hospitalized at four general hospitals between March 2008 and June 2009. This study was approved by the institutional review board of VHS Medical Center, and each patient or legal guardian provided written informed consent to participate in the study.

### Clinical assessment

The clinical data of the patients during their hospitalization period was entered in the stroke registry, according to the standard guidelines to increase the credibility of the study. The stroke registry of every hospital collects patient information according to the standards and definitions of the Korean Stroke Registry, established by the participation of 29 large hospitals in the Korean Stroke Society in 2001, enabling direct comparison of the data [4]. The contents include the name of the registered hospital;personal information such as age, sex, height, and weight of the patient; and risk factors such as history of stroke, smoking, HTN, DM, hyperlipidemia, atrial fibrillation, and heart diseases that can cause embolism. The clinical manifestations include the time from onset to hospital visit, the National Institute of Health Stroke Scale (NIHSS) at admission and discharge, the subtype of the stroke according to the Trial of Org 10172 in Acute Stroke Treatment (TOAST) [5], and the modified Rankin Scale (mRS) at discharge and 3 months after onset through maintenance of a systematized phone history [6].

### Statistical analysis

We analyzed the relationship between history of AO exposure and short-term prognosis. We also compared demographics, risk factors for stroke, initial NIHSS scores, and stroke subtypes between AO exposure group and control group. Continuous or numerical variables were expressed as the mean (standard deviation) or median (interquartile range [IQR]) and were compared by using a Student’s *t*-test or Mann-Whitney *U* test. Categorical variables were analyzed by a chi-square test or Fisher’s exact test. All statistical analyses were performed with SPSS 20.0 for Windows (IBM Corp., Armonk, NY) and *P* < 0.05 was considered statistically significant.

## Result

The exposure group included Vietnam veterans, and all subjectsin the study were men. The mean age of the exposure group and the control group was 64.8 ± 4.5 years and 65 ± 4.5 years, respectively. There was no significant difference in the height or weight between the two groups. There were 49 (44.0 %) and 93 (32.3 %) DM patients, respectively, with a higher frequency in the exposure group (*P* = 0.045). There were 6 (6.6 %) and 69 (24.0 %) hyperlipidemia patients, respectively, with fewer patients in the exposure group (*P* < 0.002). There was no difference in the distribution of other risk factors such as HTN, atrial fibrillation, and history of stroke (Table 1). Comparison of the distribution of the subtypes according to the pathogenesis of stroke revealed that small vessel occlusion was the most common (36, 39.6 %), followed by large-artery atherosclerosis (26, 28.6 %). In the control group, large-artery atherosclerosis (120, 41.7 %) was the most common, followed by small vessel occlusion (73, 25.3 %). There was a significant difference in the distribution of subtypes (*P* = 0.014) (Table 2). The NIHSS of the exposure group on admissionshowed lower scores compared to the control group (median values, 2 and 4, respectively; *P* = 0.003). There was no significant difference between the two groups in the NIHSS at discharge. The median mRSwas 1 for the exposure group and 2 for the control group, at discharge and after 3 months, thereby showing better prognosis forthe exposure group. After 3 months of discharge, there was no significant difference in the number of patients showingan ‘independent’ outcome below mRS 2 (exposure group, 63 [69.2 %], control group, 171 [59.4 %]); however, 55 patient (60.4 %) in the exposure group and 171 (59.4 %) in the control group showed an ‘excellent’ outcome below mRS 1; a higherfrequency was observed in the exposure group (*P* = 0.001) (Table 3).

**Table 1:** Comparison of the vascular risk factors

	AO(+) *N* = 91	AO(-) *N* = 288	*P*
Male	91 (100 %)	288 (100 %)	
Age	63.28 ± 7.9	67.61 ± 4.4	0.665
Hypertension	60 (65.9 %)	208 (72.2 %)	0.290
Diabetes	40 (44.0 %)	93 (32.3 %)	0.045
Hyperlipidemia	6 (6.6 %)	69 (24.0 %)	<0.001
Atrial fibrillation	9 (9.9 %)	49 (17.0 %)	0.132
Smoking	52 (57.1 %)	89 (30.9 %)	<0.001
History of stroke	12 (13.2 %)	76 (26.4 %)	0.010
Alcohol	41 (45.1 %)	130 (45.1 %)	1.000

Data are expressed as a number (percent), or mean ± standard deviation

*AO* Agent Orange

**Table 2:** Distribution of stroke subtype

Stroke subtype	AO (+) *N* = 91	AO (-) *N* = 288	*P* = 0.014
SVO	36 (39.6 %)	73 (25.3 %)	
LAA	26 (28.6 %)	120 (41.7 %)	
CE	9 (9.9 %)	46 (16.0 %)	
Others	20 (22.0 %)	49 (17.0 %)	

*SVO* small vessel occlusion, *LAA* Large artery atherosclerosis, *CE* cardioembolism

**Table 3:** Comparison of the short-term prognosis

	AO(+) *N* = 91	AO(-) *N* = 288	*P*
Initial NIHSS	2 (1–5)	4 (2–7)	0.003
NIHSS atdischarge	2 (1–5)	3 (1–5)	0.483
mRSat discharge	1 (1–3)	2 (1–3)	0.026
mRS at 3 months	1 (0–3)	2 (1–3)	0.024
Excellent outcome (mRS 0–1)	55 (60.4 %)	118 (41.0 %)	0.001
Independent outcome (mRS 0–2)	63 (69.2 %)	171 (59.4 %)	0.092

*NIHSS* National Institutes of Health Stroke Scale, *mRS* modified Rankin Scale

## Discussion

AO and TCDD are not synonymous, but AO toxicity is thought to be caused mainly by TCDD [7].

The study of the mortality patterns in New York State Vietnam Veterans was the first study that discussed the correlation of cardiovascular diseases with TCDD, where the cross ratio of cardiovascular diseases including stroke was as high as 1.48(1.19–1.83) [8].In epidemiological studies published in the late 1990s, several authors reported that TCDD contributes to the occurrence of IHD [1],and previous animal studies have shown that TCDD leads to decreased heart function and deformation of the structure [9].In 2004, an animal study proved that dioxin increased hyperlipidemia, blood pressure, and heart muscle weight. [10]Many other studies have reported that TCDD causes DM [11].

According to the “Veterans and agent orange: update” published by the Institute of Medicine at the National Academy of Sciences, DM has been classified as a suggestive category that is possibly caused by AO since the 2000 update, and since the 2006 update, HTN has been classified as such, while IHD has been classified as such since the 2008 update [2, 12].

Stroke has been classified as a suggestive category since the 2014 update, based on the fact that its pathogenesis is similar to IHD, and it shares similarvascular risk factors, based on recent epidemiological studies on similar substances [3, 13, 14].However,there are limited studies that verify the correlation between stroke and AO, while no studies have been conducted in Vietnam veterans,which could provide sound evidence.

Further, strokes are divided into subtypes, such as hemorrhagic strokes, small vessel disease, and cardio-embolic strokes,according to the pathogenesis, and this is much more complicated than IHD,which mostly originates from artherosclerosis. We are yet to ascertain the correlation between AO and stroke.

In this study, various clinical manifestations were compared according to the history of AO exposure to identify any differences, and several differences were confirmed. The exposure group had higher DM and less hyperlipidemia; this was ascertained while comparing the vascular risk factors.

There was no difference in other risk factors such as HTN, smoking, and history of stroke. The exposure group was expected to have more atrial fibrillation, based on previous studies that showed that AO could induce structural changes in the heart, although the sample sizes were small. There was also some difference in the subtype distribution. The exposure group was expected to have a higher frequency of large vessel disease, which occurs from artherosclerosis similar to IHD, or cardio-embolic stroke, which occurs secondary to impaired heart function, butthe frequency of small vessel disease was the highest. As a result, the short-term prognosis of the exposure group was better when comparing NIHSS at discharge or mRS after 3 months. In exposure group, small vessel disease frequency is higher. Small vessel disease is known to low association with hyperlipidemia, Sothere can be little association with hyperlipidemia and AO exposure. If small vessel disease is more common in the AO exposure group, additional studies are necessary to investigate the possibility of toxicity affecting small vessels or toxicity specific to brain tissue, in addition to previously known mechanisms.

This study has many limitations. AO exposure was confirmed in Vietnam veteran patients, but accurate information of the amount or duration of exposurewas not obtained, as in other veteran-based studies. Patients with a history of AO exposure were all registered at the same institution. We were not able to determine whether the AO exposure directly affected the occurrence of stroke or the increasedrisk, asonlythe clinical manifestations in stroke patients were compared.

Despite the limitations, this study targeted patients who are Vietnam veterans, thereby confirming a difference in the distribution of vascular risk factors and short-term prognosis according to AO exposure. This difference in clinical manifestations suggests that AO exposure has contributed to a certain extent to the onset of stroke. Larger systematic studies will be necessary in the future.

## Conclusion

This study targeted patients who are Vietnam veteran. There is some difference in vascular risk factors and clinical manifestations suggest AO exposure has contributed to a certain extent to the stroke. The exposure group frequency of small vessel disease was the highest and the short-term prognosis of the exposure group was better when comparing NIHSS at discharge or mRS after 3 months. It is needed to study more systematically and with larger scale in the future.
